# Mineralized Collagen: Rationale, Current Status, and Clinical Applications

**DOI:** 10.3390/ma8084733

**Published:** 2015-07-24

**Authors:** Zhi-Ye Qiu, Yun Cui, Chun-Sheng Tao, Zi-Qiang Zhang, Pei-Fu Tang, Ke-Ya Mao, Xiu-Mei Wang, Fu-Zhai Cui

**Affiliations:** 1 School of Materials Science and Engineering, Tsinghua University, Haidian District, Beijing 100084, China; E-Mails: ye841215@gmail.com (Z.-Y.Q.); cuifz@mail.tsinghua.edu.cn (F.-Z.C.); 2 Beijing Allgens Medical Science and Technology Co., Ltd., No.1 Disheng East Road, Yizhuang Economic and Technological Development Zone, Beijing 100176, China; E-Mails: cuiyun@allgensmed.com (Y.C.); zhangzq@allgensmed.com (Z.-Q.Z.); 3 The 401 Hospital of Chinese People’s Liberation Army, No. 22 Minjiang Road, Qingdao 266071, China; E-Mail: taocsqingdao@gmail.com; 4 The General Hospital of People’s Liberation Army, No. 28 Fuxing Road, Beijing 100853, China; E-Mails: tangpeifu301@gmail.com (P.-F.T.); maokeya@sina.com (K.-Y.M.)

**Keywords:** mineralized collagen, collagen, hydroxyapatite, biomineralization, self-assembly, medical device product, clinical application

## Abstract

This paper presents a review of the rationale for the *in vitro* mineralization process, preparation methods, and clinical applications of mineralized collagen. The rationale for natural mineralized collagen and the related mineralization process has been investigated for decades. Based on the understanding of natural mineralized collagen and its formation process, many attempts have been made to prepare biomimetic materials that resemble natural mineralized collagen in both composition and structure. To date, a number of bone substitute materials have been developed based on the principles of mineralized collagen, and some of them have been commercialized and approved by regulatory agencies. The clinical outcomes of mineralized collagen are of significance to advance the evaluation and improvement of related medical device products. Some representative clinical cases have been reported, and there are more clinical applications and long-term follow-ups that currently being performed by many research groups.

## 1. Introduction

Mineralized collagen is the building block for various connective tissues such as bone, cartilage, tendon, and dentin [1,2,3]. The orderly, organized mineralized collagen with a unique nanostructure constitutes the foundation for a series of physiological functions of the connective tissues. Extracellular bone matrix contains 60 wt % to 70 wt % mineral components in the form of nano-sized apatite crystals, 20 wt % to 30 wt %collagen fibers, and 10 wt % to 20 wt % water [4]. The main component of the mineral is hydroxyapatite (HA, Ca_10_(PO_4_)_6_(OH)_2_), with a stoichiometric Ca/P ratio of 1.67. Actually, the HA within the natural bone tissues are commonly the Ca-deficient–type with the Ca/P ratio lower than 1.67 [5]. The main fibrous protein is type I collagen, which is a major component of the natural extracellular bone matrix. The fine structure of the mineralized collagen has been intensively studied and a relevant structural model has also been suggested [6]. Beyond mineralized collagen, there are several higher levels of featured assembled structures which constitute various bone tissues with different physiological functions and biomechanical properties [7].

Utilizing biomimetics as a guiding principle in developing new materials, synthesizing bone substitute materials mimicking natural mineralized collagen has been attracting interests from researchers in the field of biomaterials [8,9,10]. Many methods were developed to prepare so-called “mineralized collagen” that consisted of collagen/hydroxyapatite (Col/HA) composites with or without the self-assembly structure of the natural bone mineralized collagen. Some of the products have been commercialized as medical devices and used to repair bone defects. Other than traditional synthetic bone grafts that only act as structural replacements, the mineralized collagen is bioabsorbable and osteoconductive and could promote bone regeneration.

In this paper, we review the rationale for mineralized collagen, summarize the current status of mineralized collagen medical device products, and introduce the clinical applications of such biomimetic Col/HA composites for bone defect repair in many clinical indications.

## 2. Rationale for Mineralized Collagen

Collagen is an important family of proteins for the vertebrate [11]. More than 20 types of collagens have been discovered in previous studies, and the most abundant for human beings is type I collagen [12]. The triple helix features and polypeptide chain characteristics for the primary structure of type I collagen has been investigated in-depth and systematically reviewed [13,14]. The collagen is synthesized inside the cells, then extruded to extracellular space where it undergoes fibrillogenesis, and is followed by self-assembly before the mineralization [1].

Natural bone tissues are complex biomineralized systems with hierarchical structures, and the elementary unit is mineralized collagen, which is composed of orderly, organized collagen and HA [15]. There are different hypotheses on the rationale for mineralized collagen. One hypothesis puts forth that the collagen is mineralized during bone formation with a process of polymer-induced liquid-precursor (PILP) [16,17]. In such a PILP process, a fluidic amorphous liquid-phase mineral precursor to the HA is generated, and the fluidic character enables the amorphous precursor to be drawn into the nano-scale gaps and grooves of collagen fibrils by capillary action, so as to facilitate intrafibrillar mineralization of type I collagen. The amorphous precursor then solidifies and crystallizes upon loss of hydration waters into the more thermodynamically stable phase, leaving the collagen fibrils embedded with nano-sized HA crystals [16]. Another hypothesis proposes that the mineralized collagen forms via a self-assembly process that HA crystals deposit in an orderly fashion on the nucleation sites within type I collagen fibrils at ambient temperatures [18]. In the mineralization process of mineralized collagen, nucleation sites are of importance for the formation of the mineral crystals. Previous studies suggested that the negatively charged carboxyl groups (–COOH), which are present in about 11% of the amino acid residues of collagen molecules, are the major nucleation sites for the mineralization of collagen fibrils, and the binding of calcium ions (Ca^2+^) on these carboxyl groups is a key factor for the initial step of the crystal nucleation [19]. A subsequent study indicated that not only the carboxyl group, but also the carbonyl groups (C=O) on collagen serve as another kind of nucleation site for the mineralization of the HA crystals [20]. The oxygen atoms of both types of groups coordinate with Ca^2+^ as the core of heterogeneous nucleation, and then crystals nucleate and grow. The mineralized collagen fibrils further assemble to form a matrix with a three-dimensional net structure as a template for further mineralization. Within the mineralized collagen, the conformation of the collagen protein changes and collagen fibrils cross-link through coordination bonding between saccharide hydroxyl and Ca^2+^, thus making the collagen undissolvable in water [21,22]. The assembly and organization of the collagen provides intramolecular space for the nucleation and growth of the HA crystals [23]. At the initial stage, the nucleation occurs within the gap and overlap regions created in the assembly of type I collagen molecules [6,24]. Once nucleated, the HA crystals develop into small platelets, and grow in the direction of the crystallographic *c*-axis as a preferred orientation along and parallel to the long axis of a collagen fibril [18]. Besides the gap and overlap regions, mineralization on the surface of the collagen fibrils was also observed and confirmed in vertebrates [25,26,27]. 

**Figure 1:**
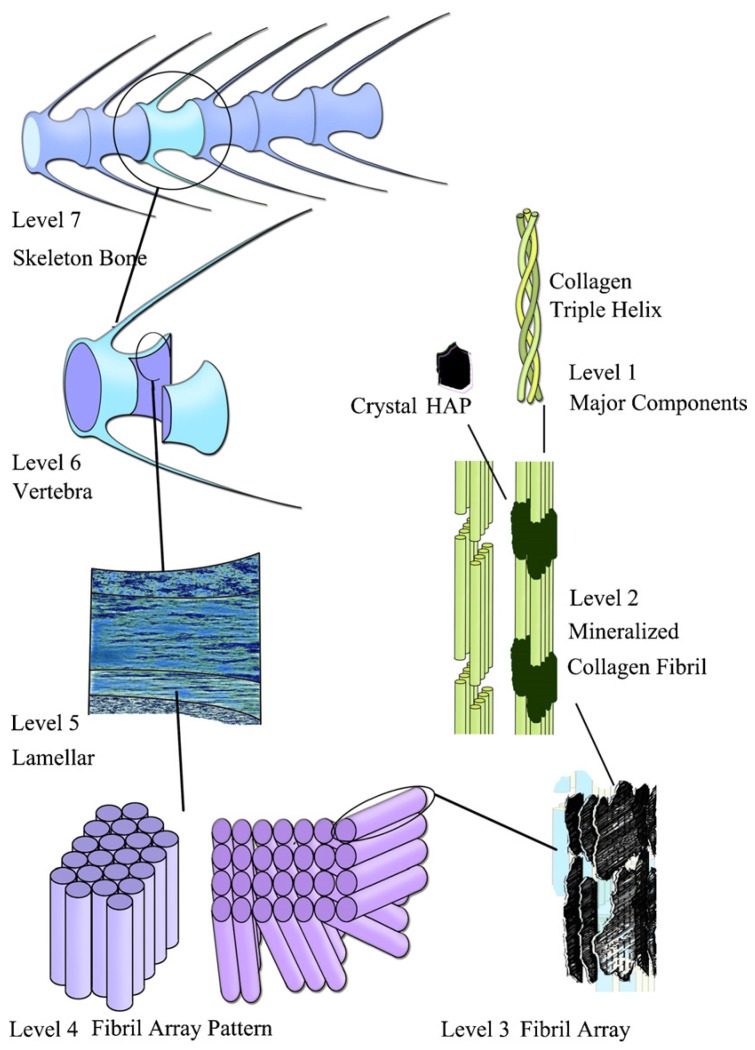
The seven hierarchical levels of organization of the zebrafish skeleton bone. Reprinted with permission from reference [1]. Copyright 2007 Elsevier.

Several models for higher level structures of the bone tissues have been suggested. According to a hierarchical organization of human long bone described by Weiner *et al.*, a complex structure with seven hierarchical levels was proposed [28]. As seen in the schematic diagram of hierarchical levels of the zebrafish skeleton bone shown in Figure 1, Level 1 is HA crystals and collagen fibrils; Level 2 is the mineralized collagen fibril; Level 3 is the array of mineralized collagen fibrils with a cross-striation periodicity of nearly 60–70 nm; Level 4 is two fibril array patterns of organization as found in the zebrafish skeleton bone; Level 5 is the lamellar structure in one vertebra; Level 6 is the vertebra; and Level 7 is the skeleton bone [29]. For other vertebrates, there might be four fibril array patterns of organization in Level 4 [28]. Although there are different criteria for describing the hierarchical structures of the bone tissues, the basic structural unit is the mineralized collagen [30,31,32].

## 3. Studies on Biomimetic Mineralized Collagen 

Based on the understanding of natural mineralized collagen and its formation process, many studies have been performed to prepare biomimetic materials mimicking natural mineralized collagen [33,34,35]. Until now, there have been a number of bone substitute materials developed via different methods based on mineralized collagen. Some of these materials have been commercialized and approved by governmental administrations as medical device products.

Kikuchi *et al.* [36,37] synthesized an HA and collagen composite, using Ca(OH)_2_, H_3_PO_4_, and porcine atelocollagen as staring materials for a self-assembly mechanism through a simultaneous titration method under controlled pH and temperature. Under the condition of 40 °C and pH = 8, they prepared a material with bone-like inorganic and organic composition.

Constantz *et al.* [38] disclosed a preparation method for mineralized collagen in U.S. Patent No. 5,231,169. The method employs preparing calcium phosphate *in situ* in a dispersion of collagen fibrils at high pH value. A source of soluble calcium and a source of soluble phosphate are then gradually and continuously added to the collagen-fibril-containing medium at an elevated pH value as high as 11–12 to form calcium phosphate while incorporating other components into the crystal lattice. 

Bradt *et al.* [39] studied the mineralization of collagen by combining the assembly of collagen fibrils and the formation of calcium phosphate in one step. By mixing an acidic calcium-containing collagen solution with a phosphate-containing neutralization buffer, the two reactions both started simultaneously. At the very beginning, the amorphous calcium phosphate phase occurred along with precipitation and then transformed into a crystalline apatite-like phase. In this way, a homogeneously mineralized collagen gel consisting of a three-dimensional network of collagen fibrils covered with calcium phosphate was obtained. Moreover, polyaspartate was added to the reaction mixture to improve the connections between the collagen fibrils and the calcium phosphate crystals. 

Pederson *et al.* [40] exploited temperature-sensitive Ca- and P-loaded liposomes to trigger Ca^2+^ and PO_4_^3−^ release, and combined with thermal collagen gelation via self-assembly to form a mineralized collagen composite, thereby mimicking the processes of natural mineralized tissue formation.

Olszta *et al.* [16] proposed that the high degree of intrafibrillar mineralization during bone formation was achieved by capillary action applied to a fluidity of an amorphous precursor that is induced by the highly anionic non-collagenous proteins of the bone matrix. By using carboxylate-rich biomimetic polypeptides, they created a synthetic composite mimicking the nanostructure of bone that nano-sized HA crystals oriented in the *c*-axis along the long axis of the collagen fibril.

Ficai *et al.* [41] prepared a Col/HA composite material through a self-assembling method, which started from collagen gel and HA precursors in conditions of 37 °C, pH = 9, and air drying by an *in vitro* mineralization method. HA was obtained by co-precipitation in the presence of collagen gel. In this way, oriented Col/HA nano-composite materials can be obtained through a simple and achievable method, the average orientation degree in this material being 97.46%.

Maas *et al.* [42] designed and built a U-tube setup consisted of two half U-tubes and a nanoporous membrane sandwiched between the two halves. Before synthesis, raw materials divided into two groups were filled into the two sides of the nanoporous membrane. One group was acidic collagen solution with Ca^2+^, the other solution contained Na_2_HPO_4_ and NaOH. The collagen with Ca^2+^ was then pressed to get through the membrane and mineralization occurred on the collagen fibrils. The product was demonstrated to be mineralized collagen with amorphous minerals.

Wang *et al.* [43] fabricated a biomimetic Col/HA composite scaffold with a three-dimensional porous structure by using a microwave-assisted *in situ* co-precipitation processing route. Co-titration of phosphorous acid-containing collagen solution and calcium hydroxide-containing solution were simultaneously added into a reaction vessel through a pump at a predefined rate and at 40 °C and pH = 9. The collagen fibril formation and HA formation could be achieved in one process step. During the co-precipitation process, the collagen fibrils formed as templates for the precipitation of HA crystallites.

**Figure 2:**
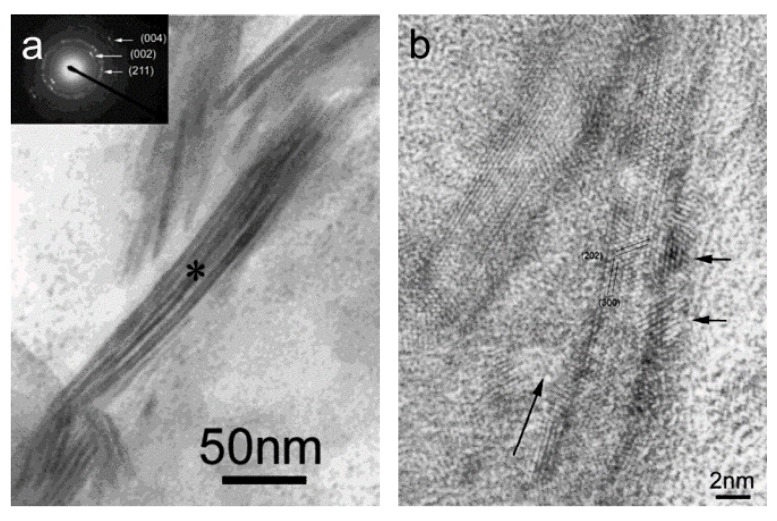
(**a**) Higher magnification of the mineralized collagen fibrils. Insert is the selected area electron diffraction pattern of the mineralized collagen fibrils. The asterisk is the center of the area and the diameter of the area is about 200 nm. (**b**) High-resolution transmission electron microscopy (HR-TEM) image of mineralized collagen fibrils. Long arrow indicates the longitude direction of the collagen fibril. Two short arrows indicate two hydroxyapatite (HA) nanocrystals. Reprinted with permission from reference [44]. Copyright 2003 American Chemical Society.

**Figure 3:**
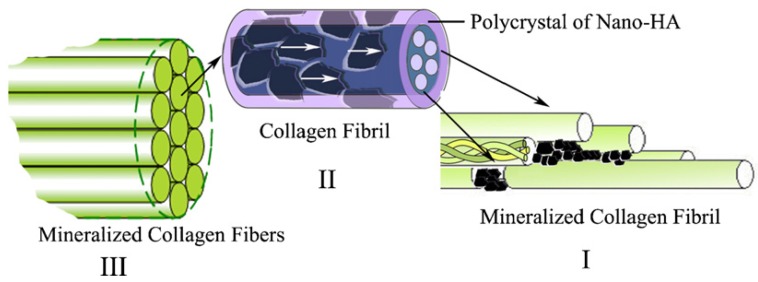
The hierarchical structure of the self-assembled mineralized collagen. (In **I**, the first level, the organization of the collagen molecules with the nano-sized HA crystals formed initially in the gap zones between the collagen fibrils; in **II**, the second level, showing the organization of collagen fibrils with respect to HA crystals, the HA crystals are platelet-like and grow on the surface of these fibrils in such a way that their *c*-axes are oriented along the longitudinal axes of the fibrils, as indicated by the white arrows in the figure; in **III**, the third level, showing the organization of the mineralized collagen fibrils, a number of mineralized collagen fibrils align parallel to each other to form mineralized collagen fibers. Reprinted with permission from reference [1]. Copyright 2007 Elsevier.)

**Figure 4:**
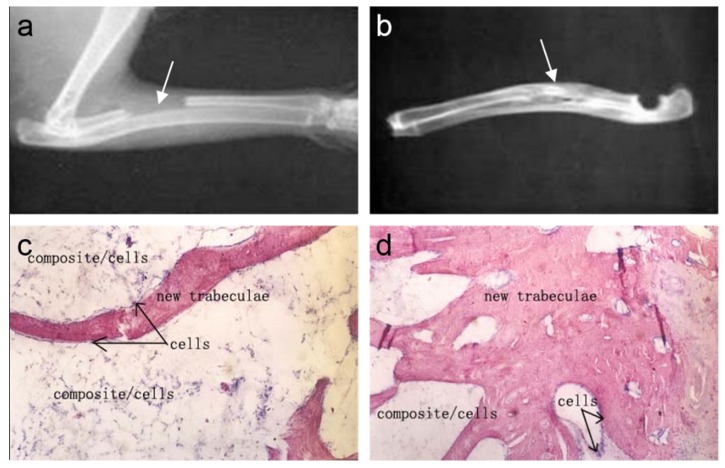
Repair of a critical defect on the rabbit radius by using mineralized collagen: (**a**) After surgery (the defect is noted by the arrow). (**b**) Implant at 12 weeks, double cortical bone connected completely (the regenerated bone tissue is noted by the arrow). (**c**) Implant at eight weeks, decalcified histology hematoxylin-eosin staining. (**d**) Implant at 12 weeks, decalcified histology hematoxylin-eosin staining (compared to eight weeks, more new trabeculae replace the composite). Reprinted with permission from reference [49]. Copyright 2004 John Wiley and Sons.

Cui *et al.* [44] designed and prepared biomimetic mineralized collagen nano-fibrils on the basis of a large number of their previous studies [45,46,47]. This biomimetic mineralized collagen was similar to the natural bone in terms of both the composition and the nanostructure. During the preparation of the composite, HA crystals grew on the surface of the collagen fibrils with triple helices and their crystallographic *c*-axes oriented along the longitudinal axes of the fibrils. The mineralized collagen fibrils aligned parallel to each other to form mineralized collagen fibers. For the first time, the new hierarchical self-assembly structure of the collagen-HA composite was verified by conventional and high-resolution transmission electron microscopy (HR-TEM), as shown in Figure 2 [44]. It could be clearly seen that the nanocrystals of the HA minerals were deposited along the surface of the fibrils, which is considered to be direct evidence to support previous biomineralization theories [48]. Figure 3 shows a schematic diagram of the hierarchical structure of the self-assembled mineralized collagen composite [1]. Furthermore, Liao *et al.* [49] used rabbits with a 15 mm segmental defect in the radius as a model to evaluate the bone-remodeling ability of this biomimetic composite. The results shown in Figure 4 demonstrated that the biomimetic mineralized collagen repaired the critical defect on the long bone (Figure 4a,b) [49]. Tissue slices show that along with the degradation of the material, the implanted composite was substituted by new trabeculae (Figure 4c,d) [49]. 

Non-collagenous proteins also play important roles in collagen mineralization by controlling mineral deposition, orientation, and phase [50]. Therefore, non-collagenous proteins were also involved in biomimetic preparation of the mineralized collagen. 

Olszta *et al.* [16] successfully performed intrafibrillar mineralization of the collagen by using the process of PILP. Similar to the natural bone, the HA crystallites were preferentially aligned with [001] orientation along the collagen fibril axes within the mineralized collagen. This study was accomplished by using polyaspartate (polyAsp) to stabilize a precursor phase of amorphous calcium phosphate (ACP), which is highly hydrated, thus facilitating its infiltration into the interstices of the fibrils.

Deshpande *et al.* [51] used polyAsp as analog non-collagenous acidic proteins to mimic the mineralized collagen fibrils; transmission electron microscopy and electron diffraction showed that the mineral crystals in the fibrils were organized into arrays with their *c*-axes co-aligned with the long axes of the fibrils, indicating that the influence of polyAsp to the mineralization process is similar to acidic non-collagenous proteins in bones and dentin. 

Wang *et al.* [52] designed and synthesized a polypeptide with sequence (EEEEEEEEDS_p_ES_p_S_p_EEDR) to mimic the function of bone sialoprotein and dentine matrix protein 1 as calcium phosphate- and collagen-binding matrix protein analogues. In the study of *in situ* remineralization of acid-etched dentine, the synthetic peptide was found to promote the transition of calcium phosphate (CaP) nanocrystals to large apatite platelets via the formation of mesocrystal intermediates.

Burwell *et al.* [53] applied the PILP system to artificial lesions to achieve functional remineralization of carious dentin lesions. PolyAsp with 27 kDa was used as the polymeric process-directing agent, and was added to the remineralization solution containing calcium/phosphate with a molar ratio of 2.14. During the four weeks of treatment, functional remineralization of partially demineralized human dentin occurred with the recovery of mechanical properties. With progressive intra- and extra-fibrillar mineralization initiated in the depth of the lesion, the degree of remineralization was gradually increasing.

Antebi *et al.* [54] used the PILP process in conjunction with a perfusion-flow system to establish a dynamic PILP process to fabricate porous Col/HA composite. The mineralization was performed using a Tris-based mineralization solution containing K_2_HPO_4_, CaCl_2_, and polyAsp at 37 °C. The perfusion-flow system was created via a stainless steel mold to direct the mineralization solution flow through the center of a porous collagen scaffold. In comparison with the static mineralization method, this study revealed that the dynamic technique facilitated more efficient and homogenous mineral deposition throughout the Col/HA composite.

## 4. Commercially Available Mineralized Collagen Products

Compared to traditional bone grafts made of metals or ceramics, mineralized collagen possesses many advantages, such as biomimetic composition, biodegradation, better biocompatibility, and better biomechanics [55,56]. With the increasing acceptance of biomimetic materials, there have been several Col/HA products approved by governmental administrations and gradually recognized and adopted by surgeons. Table 1 summarizes Col/HA medical device products on the market, such as HEALOS^®^ (Johnson & Johnson, New Brunswick, NJ, USA), Vitoss^®^ (Stryker, Kalamazoo, MI, USA), OSTEON^®^ (Dentium, Suwon, Korea), and so on. These products are commonly intended to be used in the treatment of osseous defects caused by traumatic injury, tumors, surgical wounds, and so on. Although these composite materials are composed of collagen and calcium phosphate ceramics, their composition, pore size, porosity, indications, and characteristics may differed from each other. 

HEALOS^®^ Bone Graft Replacement is a mineralized collagen matrix processed into lyophilized strips or pads for surgical implantation. The principal components of HEALOS^®^ are type I bovine collagen and HA, and the component of mineral is approximately 30% by weight. The collagen is processed prior to mineralization using aqueous and organic purification steps to reduce lipids, salts, and endotoxins. HA is coated on the surface of the collagen fibers by the controlled addition of calcium chloride, sodium phosphate, and sodium hydroxide. The mineralized collagen fibers are fixed into a three-dimensional, open-cell matrix, and the porosity is higher than 95% and the pore size is about 4–200 μm, and can be fully resorbed during the natural process of bone formation and remodeling [57,58].

Vitoss^®^ Foam Bone Graft Substitute is made of calcium phosphate and type I bovine collagen. The products can be provided in forms of cylinder, strip, putty, *etc.* It is an osteoconductive porous implant with a trabecular structure that resembles the multidirectional interconnected porosity of human cancellous bone. Pore diameters in the scaffold range from 1–1000 μm. Vitoss^®^ Bone Graft Substitute is bioresorbable, and guides the regeneration of bone and other connective tissues in the defect site into the space it is implanted [58].

**Table 1:** Commercially available mineralized collagen products.

Product	Company	Composition	Porosity	Pore Size	Form	Claimed mechanisms of action
Bio-Oss Collagen^®^	Geistlich, Switzerland	10% type I collagen and 90% bovine mineral	70%–75%	300–1500 μm	Block	Osteoconduction
Bongold^®^	Allgens, China	Self-assembled type I bovine collagen and hydroxyapatite (HA) similar to the natural mineralized collagen	70%–88%	50–550 μm	Strip, granule, block, putty, sponge	Osteoconduction Bioresorbable Osteogenesis and osteoinduction when mixed with bone marrow aspirate
CopiOs^®^	Zimmer, USA	Type I bovine collagen and 67% mineral	93.39%	-	Sponge, paste	Osteoconduction Bioresorbable Osteogenesis and osteoinduction when mixed with bone marrow aspirate
HEALOS^®^	Johnson & Johnson, USA	70% type I bovine collagen and HA	> 95%	4–200 μm	Strip	Osteoconduction Creeping substitution Osteogenesis when mixed with bone marrow aspirate
MOZAIK^™^	Integra, USA	20% type I collagen and 80% β-TCP	-	12–350 μm	Strip, putty	Osteoconduction Bioresorbable
MASTERGRAFT^®^ Strip/Putty	Medtronic, USA	Bovine type I collagen and biphasic ceramics (15% HA and 85% β-TCP)	89%	-	Strip	Osteoconduction Bioresorbable
OSTEON^™^	Dentium, Korea	8% type I collagen and 92% mineral (30% HA and 70% β-TCP)	70%	500–1000 μm	Cylinder	Osteoconduction Bioresorbable
OssiMend^™^	Collagen Matrix, USA	45% bovine type I collagen and 55% synthetic calcium phosphate	-	-	Strip, block, putty	Osteoconduction Bioresorbable
Refit	HOYA, Japan	20% type I bovine collagen and 80% HA	95%	100–500 μm	Block	Osteoconduction Bioresorbable
SynOss^™^ Putty	Collagen Matrix, USA	Type I collagen and carbonate HA	-	-	Putty	Osteoconduction
Vitoss^®^ FOAM	Stryker, USA	Type I bovine collagen and calcium phosphate	90%	1–1000 μm	Putty, strip, flow, morsels and shapes	Osteoconduction Bioresorbable Osteogenesis and osteoinduction when mixed with bone marrow aspirate

CopiOs^®^ Bone Void Filler Sponge (Zimmer, Warsaw, IN, USA) is a synthetic bone graft material consisting of calcium phosphate and type I bovine collagen sponges by a vacuum freeze-dry method. The sponge contains approximately 67% mineral by weight. CopiOs^®^ is claimed to provide a moderately acidic environment that promotes solubility of osteoinductive growth factors such as bone morphogenetic proteins. The average porosity of the product was determined to be 93.39%, and the scaffold is bioresorbable, thus possessing an osteoconductive capacity [59].

MASTERGRAFT^®^ Strip and Putty (Medtronic, Minneapolis, MN, USA) are resorbable, malleable, osteoconductive scaffolds composed of biphasic calcium phosphate (15% HA and 85% β-tricalcium phosphate (β-TCP)) ceramic granules and purified fibrillar bovine type I collagen. The porosity of the product is as high as 89% [60,61,62].

OSTEON^™^ II Collagen is a material composed of 92% synthetic calcium phosphate (OSTEON^™^ II, a mixture of 30% HA and 70% β-TCP) and 8% type I collagen. OSTEON^™^ II is claimed to be highly resorbable due to higher β-TCP content, while collagen content will be resorbed over several weeks after the initial shaping. OSTEON^™^ II Collagen is intended to be used for dental applications, including ridge augmentation, extraction socket grafting, cystic cavities, and periodontal defects. The product has a moldable property, so that it can accommodate any shape or form [63].

Bongold^®^ Bone Graft Material (Allgens, Beijing, China) is a composite of synthetic non-ceramic HA and type I collagen. It contains approximately 45% mineral by weight. The composite material of Bongold^®^ is synthesized via an *in vitro* biomimetic process similar to the mineralization of natural bone, and the products possess composites and nanostructure similar to natural mineralized collagen [64]. Bongold^®^ products are provided in many forms, such as strip, granule, block, putty, and sponge. Its porosity is greater than 70%, and the pore size is 300 ± 250 μm, thus providing a favorable environment for cell activities [56]. The biomimetic Bongold^®^ is bioresorbable and osteoconductive for the regeneration of bone tissues [49,65,66].

## 5. Clinical Applications of Mineralized Collagen Products 

Although there were few commercially available mineralized collagen products until now, remarkable clinical outcomes have been achieved by these products. Since the bone tissues exist throughout the whole human body, the biomimetic Col/HA composite could be widely used for the repair of a variety of bone defects at different sites [67]. In this section, the clinical applications of mineralized collagen products are reviewed in terms of three main departments: orthopedics, stomatology, and neurosurgery.

### 5.1. Restoration of Bone Defects in Orthopedic Applications

Bone defect repair is the main application of mineralized collagen products. Previously published literature reported many clinical applications including bone repair in the spine and joints, defects due to trauma, and so on.

Spinal fusion surgeries require bone implants to achieve bone fusion between two adjacent vertebral bodies. Such bone implants are called the spinal fusion cage, and have commonly been made of Ti alloy, stainless steel, or polyetheretherketone (PEEK). Bioresorbable mineralized collagen was used for spinal fusion at the cervical vertebra [66]. In this case, the spinal cord of the patient was compressed by the C5-6 and C6-7 discs. A wedge of the mineralized collagen with a pore size of 100–300 μm and about 90% porosity was placed into the C5-6 space, and a metal cage was placed into the C6-7 space. Due to its high porosity, the mineralized collagen showed a low density. Lateral X-ray film at 14 weeks after the surgery shows that both C5-6 and C6-7 achieved spinal fusion, indicating the good bone fusion ability of mineralized collagen. Furthermore, the mineralized collagen was absorbed and replaced by new-born bone tissues with relative high densities. Therefore, the adjacent vertebral bodies could achieve osseous fusion by using bioresorbable mineralized collagen without any foreign matter permanently left inside the patient body. The cervical spinal fusion rate when using mineralized collagen was similar to the rates when using an autologous iliac crest bone graft [66].

As a biomimetic scaffold with components and a nanostructure similar to natural bone tissue, mineralized collagen should be an adequate scaffold for *in vivo* tissue engineering that recruits, programs, and disperses autologous cells, as well as their extracellular matrix, for tissue regeneration [68]. For example, mineralized collagen was used for the treatment of bone nonunion, which is always an important clinical unmet need that affects bone healing [64]. A 32-year-old patient suffering from bone nonunion at the left tibia was treated with mineralized collagen. During the healing process, the fracture lines blurred and finally disappeared, and the tibia completely healed with osseous union. As a contrast, the fibula without treatment did not heal during this period [64].

Mineralized collagen products were also used for the treatment of, for example, lumbar interbody fusion [69,70], bone fractures with trabecular defects [65,71], and so on. The authors suggested that mineralized collagen achieved good clinical outcomes when compared to the autologous bone in many of the anatomical sites with osseous defect repair [65,66], while many of the autograft-treated patients suffered from persistent complications at the donor site [69,72]. Moreover, the combination of the mineralized collagen and bone marrow aspirate was considered to be an alternative to autologous bone [70,73].

### 5.2. Bone Volume Augmentation in Dental Surgeries

In dental surgeries, the height and width of the alveolar ridge will largely decrease after tooth extraction due to the resorption of surrounding bone [74,75]. In order to ensure adequate bone volume for subsequent implantation, site preservation at the extraction socket has become a popular approach to reduce the bone resorption trend [76]. Conventional bone graft material for dental applications was heterogeneous bone. For example, deproteinized bovine cancellous bone was used for site preservation at the alveolar ridge or for sinus augmentation [77,78]. 

Due to the infection risk and non-biodegradability associated with the xenograft, mineralized collagen bone grafts should be a promising alternative for increasing bone volume in oral surgeries. Preclinical trials showed that a mineralized collagen composed of biphasic calcium phosphate (HA/β-TCP) and collagen were able to stimulate new bone formation in reconstruction of deficient alveolar ridges for dogs [63,79].

Furthermore, growth factor has been attempted to be combined with the mineralized collagen to achieve better bone regeneration. For example, mineralized collagen bone substitutes were combined with recombinant human platelet-derived growth factor-BB (rhPDGF-BB) with a concentration of 0.3 mg/mL, and then implanted into extraction socket defects in patients. Evident bone formation was observed for each patient without any unanticipated adverse events [80].

In periodontal defect regeneration, the guide tissue regeneration (GTR) technique has been widely applied in clinics. Traditional GTR membranes only served as a barrier around the periodontal defect, so as to prevent the ingrowth of epithelial cells and fibroblasts, thereby maintaining a space for periodontal tissue regeneration [81]. With the material development and higher clinical requirement of the GTR, the third generation membrane product possesses not only bioresorbability, but also osteoconductivity to accelerate osseous regeneration [81]. Bone augmentation effects of such new generation GTR membranes have been proved by many animal experiments [82,83], and some products are commercially available for clinical applications [63,79].

### 5.3. Reconstruction of Skull Defects in Neurosurgical Applications

Many neurosurgical surgeries involving craniotomy resulting in bone defects to the skull. For example, one or more burr holes are left after a craniotomy due to drilling prior to taking the bone flap out. Although these bone defects were usually directly covered by scalp without repair, they may lead to further risks, such as leakage of cerebrospinal fluid or infection [84]. Moreover, the scalp depression at the defect sites also influence cosmetic outcomes after the surgery [85].

Autologous bone and many artificial bone grafts were used to repair or fill the defects on the skull [84,85,86]. However, harvesting autologous bone from the inner side of the skull flap reduces its thickness, and the amount of such an autograft is limited. Other bioceramics or non-degradable polymer implants permanently stay within the patient's skull.

In the treatment of bone defects after subarachnoid cyst removal at the left temporal region of a 12-year-old male child, four 2.0 cm × 1.0 cm bone defects were produced after the surgery. The two defects at the right side were implanted with the mineralized collagen, and the two defects on the left were blank controls. Follow-ups show that bone mineral density gradually increased and approximated to the host temporal bone in the mineralized collagen group. The interfaces between the implants and the host bone became fuzzy, thus indicated a remarkable osteogenesis effect. Meanwhile, the blank control sites remained having low bone mineral density compared to the surrounding normal bone tissues. The results will be published soon.

In repairs for the relatively large skull defect, commonly used repair materials are metals (e.g*.*, titanium mesh) or PEEK. They are non-biodegradable and would hinder new bone formation at the defect, especially for children with a growing skull. Bioresorbable mineralized collagen could be degraded and replaced by regenerated bone tissue, thus performing reconstruction of the bone defect on the skull. Previous clinical studies achieved desirable outcomes in repairing relatively large bone defects (6.0 cm × 6.0 cm) on the skull of children by using mineralized collagen [87].

## 6. Conclusions

From solo component to composite mimicking both composition and microstructure to native bone, biomimetic materials for bone tissue regeneration have been improved significantly over the past decades. Although such novel mineralized collagen materials have been commercialized and used in patients, longer follow-ups are required to determine their clinical outcomes. The mineralized collagen composites still need to be improved in many aspects, such as biomechanical properties, hierarchical structures, and biodegradation. As the rationale for fabricating mineralized collagen becomes better understood, novel biomimetic preparation routes will increasingly emerge and evolve. Accordingly, more and more mineralized collagen medical devices with better biomimetic properties and multiple functionalities will be on the market to provide better solutions for the unmet medical needs of patients. 
